# Using machine learning to study the effect of medication adherence in Opioid Use Disorder

**DOI:** 10.1371/journal.pone.0278988

**Published:** 2022-12-15

**Authors:** David Warren, Amir Marashi, Arwa Siddiqui, Asim Adnan Eijaz, Pooja Pradhan, David Lim, Gary Call, Mark Dras

**Affiliations:** 1 Macquarie University, Sydney, NSW, Australia; 2 Digital Health Cooperative Research Centre, Sydney, NSW, Australia; 3 Western Sydney University, Campbelltown, NSW, Australia; 4 Gainwell Technologies, Tysons, VA, United States of America; Vellore Institute of Technology: VIT University, INDIA

## Abstract

**Background:**

Opioid Use Disorder (OUD) and opioid overdose (OD) impose huge social and economic burdens on society and health care systems. Research suggests that Medication for Opioid Use Disorder (MOUD) is effective in the treatment of OUD. We use machine learning to investigate the association between patient’s adherence to prescribed MOUD along with other risk factors in patients diagnosed with OUD and potential OD following the treatment.

**Methods:**

We used longitudinal Medicaid claims for two selected US states to subset a total of 26,685 patients with OUD diagnosis and appropriate Medicaid coverage between 2015 and 2018. We considered patient age, sex, region level socio-economic data, past comorbidities, MOUD prescription type and other selected prescribed medications along with the Proportion of Days Covered (PDC) as a proxy for adherence to MOUD as predictive variables for our model, and overdose events as the dependent variable. We applied four different machine learning classifiers and compared their performance, focusing on the importance and effect of PDC as a variable. We also calculated results based on risk stratification, where our models separate high risk individuals from low risk, to assess usefulness in clinical decision-making.

**Results:**

Among the selected classifiers, the XGBoost classifier has the highest AUC (0.77) closely followed by the Logistic Regression (LR). The LR has the best stratification result: patients in the top 10% of risk scores account for 35.37% of overdose events over the next 12 month observation period. PDC score calculated over the treatment window is one of the most important features, with better PDC lowering risk of OD, as expected. In terms of risk stratification results, of the 35.37% of overdose events that the predictive model could detect within the top 10% of risk scores, 72.3% of these cases were non-adherent in terms of their medication (PDC <0.8). Targeting the top 10% outcome of the predictive model could decrease the total number of OD events by 10.4%.

**Conclusions:**

The best performing models allow identification of, and focus on, those at high risk of opioid overdose. With MOUD being included for the first time as a factor of interest, and being identified as a significant factor, outreach activities related to MOUD can be targeted at those at highest risk.

## Introduction

Opioid Use Disorder (OUD) and other forms of harmful opioid use have increased rapidly since the early 2000s. The associated personal, societal, and economic costs continue to rise despite efforts to reduce opioid prescriptions and improve patient outcomes. In 2019 there were over 50000 deaths due to drug overdose involving opioids within the USA, the highest number ever recorded [1].

To combat the growing epidemic, a number of treatments have been developed including counselling, lock-in programs and medications [2]. Many clinical trials have taken place over the decades to investigate the effectiveness of a number of medications for OUD [3, 4], and the overall recommendation from a report by the US National Academies that comprehensively reviewed previous studies strongly supports the use of medications in the treatment of OUD [5]. However, a recent systematic review of clinical studies on pharmacological assisted therapy for opioid addiction [6] noted that previous studies measure a diverse range of outcomes “which reflect success in arbitrary or opportune terms”: it observed that often primary outcomes are ones that are easy to measure, such as retention in treatment, but not necessarily ones that the report characterises as patient-important, such as abstinence or reduction in opioid use. The choice of outcome measures can be limited by sample size due to the rarity of some significant, patient-important events. Overdose events, for example, would be outcomes patients consider important, but are rare enough that they would not be feasible to model in an individual clinical study, or even in combinations of clinical studies. The systematic review of [6] found 60 studies that met their criteria, with a combined participant sample of 13,341 patients across all 60 studies; with that sample size, there would be many important rare events that would not be observed. This implies that analysis of larger-scale, real-world data is essential to better understand the full impact of these treatments.

To assess outcomes in practice, researchers have used large-scale insurance claims and electronic health records (EHR) databases to perform statistical analysis of risk factors in OUD [7, 8]. As an alternative to traditional statistical analysis, machine learning is an approach to working with larger-scale, real-world data that prioritises predictive accuracy over formal inferences about the population [9]. Machine learning techniques have demonstrated superior performance in the prediction of unobserved events in some contexts and are gaining increasing acceptance in medical and related fields [10–12]. In the context of OUD, researchers have recently used machine learning to predict overdose outcomes, for the most part based on prior opioid prescriptions [13–20]; these approaches focus on factors prior to and at the time of diagnosis, and do not consider the ongoing interaction between patient and treatment that can be crucial in longer term outcomes like medication adherence. Moreover, restricting modeling to cases involving prior opioid prescriptions becomes less helpful as many with OUD turn to illicit sources of opioids due to more rigorous prescribing controls and prescriber awareness of the dangers of over-prescribing prescription opioids. This supports the use of those in treatment as a base for modeling as proposed in this paper.

Machine learning could also be used to build predictive models based on adherence to treatment. Studies of other medications have demonstrated that participation in a clinical trial may increase medication adherence both during the trial and after the trial takes place when compared to real world usage [21]. This effect creates a potential bias in the observed effectiveness of treatment medications in clinical trials. Machine learning models can incorporate real-world prescription adherence metrics and characterise their relationship with patient outcomes. As adherence is a factor that can be improved (for example, [22]), identifying the importance of adherence metrics in such models allows an evaluation of the impact of improving adherence. In contrast to all of the previous work noted above, we use our machine learning models to analyse the effects of treatment.

The present paper describes predictive modelling of opioid overdose among patients diagnosed with OUD using large-scale Medicaid claims data, supplemented with region-level demographic data. To explore treatment specifically, we will make a number of departures from previous studies in this domain. We will target patients who are at the point of clinical diagnosis with OUD and those undergoing treatment, rather than patients who are at risk of addiction and overdose due to their prior opioid prescriptions and other factors but not yet formally diagnosed. To model this, we derive proportion of days covered (PDC) metrics from the claims data to estimate patient adherence to prescribed MOUD in the period following diagnosis to study the interaction between medication types, adherence metrics and patient outcomes. We use this to determine the extent to which prescription adherence metrics are valuable predictors of patient outcomes, and also to assess the potential benefit in improving PDC. We further stratify Medicaid beneficiaries into subgroups with similar overdose risks, and characterise the extent to which this risk stratification allows us to capture the preponderance of overdoses. Framing the problem in this way could facilitate the development of applications that could allow clinicians to identify patients at high risk of negative outcomes and consequently to focus outreach activities to support them.

## Related work

We have identified eight studies that have used machine learning in the context of OUD that are focused on predicting opioid overdoses or related outcomes. [13] and the subsequent [15] by the same group worked with Medicaid data, and focused on prediction on the basis of patterns of opioid prescriptions; [13] compared their machine learning-based risk stratification result with Centers for Medicare & Medicaid Services Opioid Safety Measures risk classification to demonstrate improvement in quantification of risk based on opioid prescription patterns. The same group extended these works by incorporating criminal justice data [16] and further validating the approach [17]. [18] used hospital data from across the US for predicting opioid poisoning. [14] aimed to predict opioid misuse or poisoning as well as dependence, in advance of the first opioid prescription, using Medicaid data for the US state of Rhode Island. [19] aimed to predict opioid-related overdose using statewide prescription drug monitoring program and hospital discharge data in the US state of Tennessee. [20], using data from Alberta, Canada, aimed to predict overdose risk within 30 days after an opioid dispensation on the basis of features related to opioid dispensations. Their cohorts are thus different from ours, and they do not look at MOUD adherence, the focus of our study. There are other studies that use machine learning in a related context, but for different tasks, such as the prediction of OUD on the basis of opioid prescriptions and additional features [23–25], success in OUD treatment [26], or MOUD adherence from other data [27].

Other studies do not use machine learning in looking at risks of OUD-related outcomes, and also particularly focus on risks in opioid prescription: [28] aimed to predict “aberrant behaviour” in opioid-treated patients and to validate an opioid risk prediction tool; [29, 30] aimed to predict opioid misuse for opioid prescription users; [31] compared predictive models for opioid misuse for two cohorts, the overall population from a private insurer and the subset with at least one opioid prescription; [32] aimed to identify patients at increased risk for problem opioid use, using natural language processing on clinical notes in addition to standard statistical analysis; and [33] developed a tool for assessing de novo opioid misuse or dependence, again considering a cohort with at least one opioid prescription.

While there are many studies on the beneficial effects of MOUD, surveyed in [5], most are different in focus from the present work, considering instead a range of other outcomes like effect on mortality, improved social functioning, etc. [7] looked at the association between MOUD along with behavioural health treatment and opioid relapses on Medicaid data using a Cox Proportional Hazards analysis, but do not specifically examine adherence.

## Materials and methods

### Data acquisition

The dataset we used to conduct this research is a subset of a “limited data set” consisting of Medicaid claims for 10 US states. The dataset is provided by HMS (a Gainwell Technologies company) through the Digital Health Cooperative Research Centre (DHCRC). It consists of Medicaid claims (inpatient, outpatient, dental, pharmaceutical) and a few demographic characteristics of the patients (age, sex, zip code, Medicaid enrollment periods) and their unique identifiers to link their claims across time and different settings of care and multiple episodes of enrollment. Diagnosis codes associated to the patient, procedures codes, prescription date, dosage, duration and other prescription details are among the information available in the dataset.

The dataset covers the claims across 2015 till 2021 and consists of more than 1.16 billion claims for more than 14.2 million unique individuals. For this study, we selected data from two states (state “A” and “B”) that are different from each other in terms of demographics and economy. Compared to state “A”, state “B” has a higher population (more than 6 times), higher GDP per capita, lower unemployment rate, lower risk of poverty, higher Human Development Index (HDI) and younger and more ethnically diverse population [34, 35]. In terms of the data, the dataset contains only Fee For Service (FFS) claims for state “A” and only Managed Care (MC) claims for state “B”.

### Data preprocessing

#### Cohort selection & index point

Of the nine million patient records in the two selected states, eligibility for inclusion in the cohort was determined by the presence of one or more claims where the primary diagnosis was an opioid-related disorder (F11) under the ICD-10 classification system with subsequent MOUD claims, i.e. diagnosis with, and MOUD prescriptions for, any one (or any subdivision) of:

- Opioid abuse (F11.1).
- Opioid dependence (F11.2).
- Opioid use, unspecified (F11.9).

**Fig 1:**
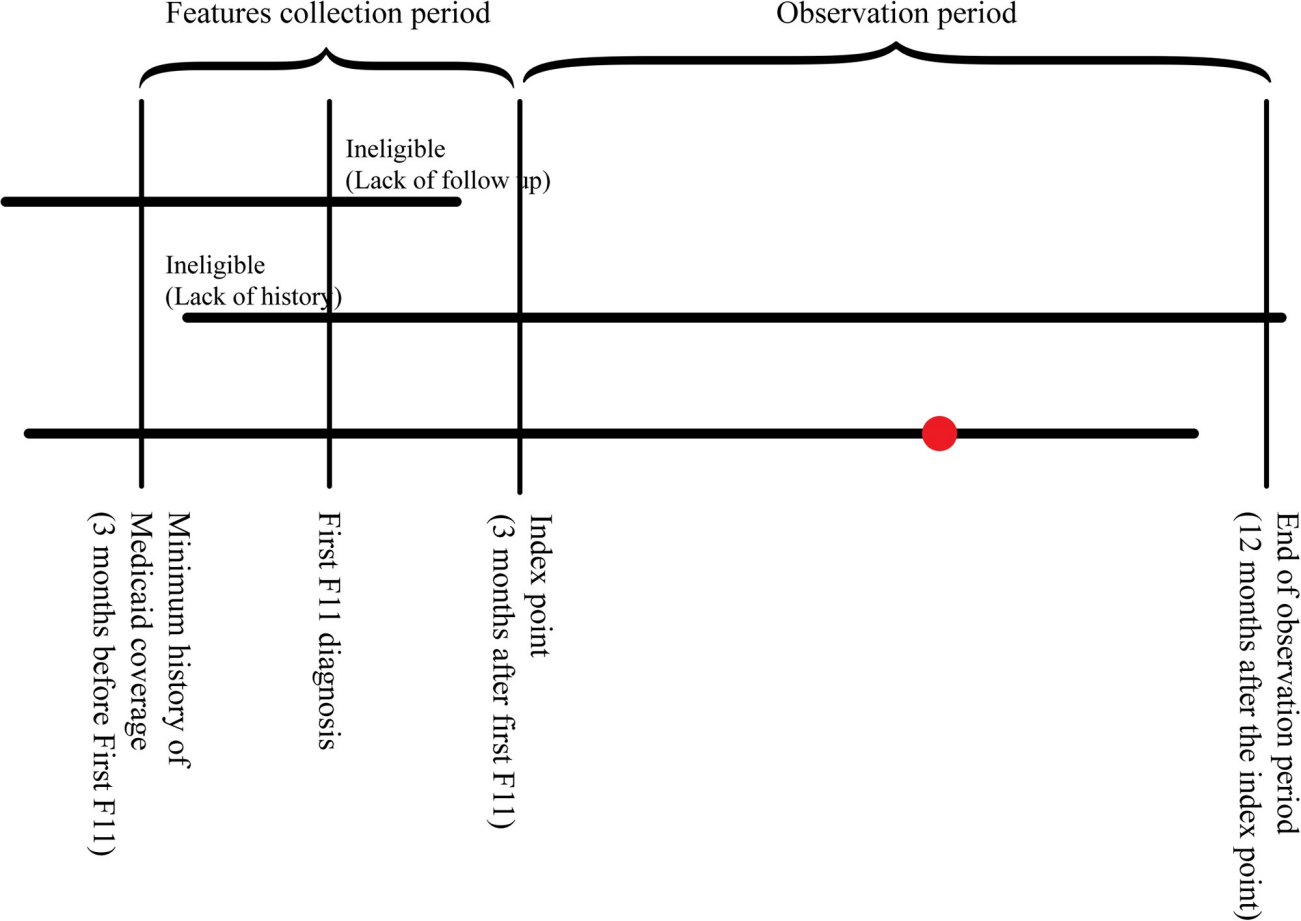
Cohort selection, patient observation timelines. Red marker indicates observed outcome event (overdose).

For our predictive model, a hypothetical ‘follow-up’ date 3 months after the first diagnosis was considered the index point. The 3-month period between diagnosis and follow-up reflects the duration of medication assisted treatment protocols used in efficacy of treatment studies [3, 36]. This index point is the time at which we would want to make predictions about the relative risk and potential outcomes for the patient based on their history and initial treatment. Thus a model makes predictions about a patient using only the data that would be realistically available at the index point. Fig 1 illustrates this setup.

The Medicaid dataset only contains claims covered under the Medicaid program, rather than a complete history of treatment for a patient. To ensure some comparability of medical history between patients, a minimum period of Medicaid coverage before the index point was required for inclusion in the cohort. Patients who became eligible for Medicaid less than 3 months before their first F11 diagnosis were removed from the study. Similarly, patients who became ineligible for Medicaid prior to their index date and would have an incomplete history of MOUD claims were removed.

#### Outcome variable: Overdose events

Overdose events were the primary outcomes of interest and were the basis for the target variable in our model. Overdose events were identified as institutional claims, including inpatient treatment and emergency room admissions, made by a patient where the primary ICD code corresponds to the category “Poisoning by, adverse effect of and underdosing of narcotics and psychodysleptics” (T40). This category covers acute adverse outcomes of drugs most relevant to the current study: overdoses by heroin (T40.1) and overdoses by other opioids (T40.2) [13].

Overdose events were divided into categories depending on the date of occurrence relative to the index point. Overdose events that occurred at or before the index point were considered independent variables for the sake of making predictions, while overdoses that occur in the 12 months following the index point were considered predictable outcomes. For simplicity, post-index overdoses were encoded as a single binary target variable for each patient indicating whether or not the patient overdosed, even if the patient had multiple post-index overdose events.

### Predictor features

#### Feature types

We used 46 different features: our key predictor of interest representing MOUD adherence (named top_pdc_3m), the dominant OUD medication (window_mat), existence of comorbidities (29 features, named prior_X), prescriptions for drugs in addition to MOUD (4 features, named ssri_rx_X and bzd_rx_X), demographic features (7 features: age, sex, income, education, employment, area type, state), history of OD (2 features: od_before_f11 and od_window_0) and history of opioid prescriptions (2 features: opd_rx_before_f11 and opd_rx_during_f11).

More details of these features are given below and in S1 Appendix, and they are summarized in Table 1. To facilitate model training, all categorical variables were one-hot encoded into a binary attribute with 1 representing the presence of that attribute and 0 representing the absence of that attribute.

**Table 1:** Summary of all model features.

feature type	# features	feature value
MOUD adherence (PDC)	1	real-valued, range [0, 1]
MOUD type	1	4 values
comorbidities	29	binary
other prescriptions	4	binary
demographic (sex, employment, state, area type)	4	binary
demographic (income)	1	3 values
demographic (age)	1	5 values
demographic (education)	1	6 values
history of OD	2	binary
history of opioid prescriptions	2	binary
**total**	46	–

**Medication adherence**. In our models, medication adherence was defined the Proportion of Days Covered (PDC) method for the OUD treatment medication (Buprenorphine, Naltrexone or Methadone). PDC is the proportion of days in a period that a patient could have taken their medication given their history of prescription claims and supplied amounts. We used PDC as a predictor variable calculated over the initial treatment period (3 first months of the treatment).

We note that a PDC score of 0.8 or more is conventionally considered adherent, and below that non-adherent. This has been the case since the 1970s [37] in the context of primary hypertension, but has since been supported empirically in a broader range of diagnosed conditions like schizophrenia and diabetes (e.g. [38]). We primarily consider PDC as a real-valued variable, but we also investigated the applicability of this kind of threshold.

**Comorbidities**. All ICD-10 diagnosis codes relating to each patient in the cohort were extracted from medical and institutional claims tables. Codes were first filtered to include only those diagnosed at or before the primary index point since these were the codes that could realistically be used to inform treatment or intervention. There are more than 77,000 unique procedure and diagnosis codes that can potentially appear in this data. To reduce the dimensionality of comorbidities, diagnosis codes were categorized into Elixhauser general comorbidity categories [39] according to the Agency for Health Research Quality (AHRQ) rules using the “icd” R package by Jack O Wasey [40]. These rules reduced the ICD-10 codes to 29 categories, with 29 corresponding binary variables in our models.

**Other prescriptions**. Similar to ICD diagnosis codes, pharmacy claims from the cohort cover a huge range of prescription medications indicated by National Drug Codes (NDC). It was not feasible to consider every possible drug, dosage and combination, therefore we relied on related work to identify a much smaller subset of medications. The clinical literature indicated 3 broad categories of medications potentially relevant to the treatment outcomes in OUD [4, 5]:

1. Selective Serotonin Reuptake Inhibitors (SSRIs) commonly prescribed for depression.
2. Benzodiazepine class drugs typically used in the treatment of anxiety and sleep disorders.
3. Opioid based analgesics.

**Demographic features**. Some demographic features of patients were drawn directly from the claims data patient eligibility table; however, this data contained very little information regarding the patient’s socio-economic status, income levels, education levels or living conditions, all of which may be valuable predictors of success. To mitigate this lack of data, we used the patient’s registered zip code to include region-level averages for many socio-economic factors as a proxy for individual data. These region-level features have been effective in other claims modelling studies [41, 42]. The Medicaid data itself includes individual patient age and sex which are encoded as categorical variables and included in all models. Zip codes of patients were used to join the patient data to supplemental region-level data on income, education, employment and urban development.

Missing region-level demographic features for two individuals without a recorded zip code were imputed using the mean calculated over all regions.

### Feature selection

We first checked for correlations between all continuous variables to prevent strong collinearities within predictors. It was found that the employment status features for employed and not in the labour force were very highly correlated (since they represent mutually exclusive sections of the population), so the latter was removed from the final dataset.

We also applied backward feature elimination to selected models as a feature selection technique to see if performance could be improved.

### Predictive model building

#### Machine learning classifiers

We used four commonly-used binary classifiers to build our models:

- Logistic Regression
- Decision Tree
- Random Forest
- Gradient boosting (XGBoost)

The classifiers predict whether there will or will not be an overdose event in the chosen time window.

All models used scikit-learn implementations configured to optimize AUC. We only selected classifiers that are able to provide a prediction score that can be interpreted as a probability of a particular outcome. As the models were not sensitive to hyperparameters, we only present results for default settings, provided in S3 Appendix.

#### Evaluation

For evaluation, the data was randomly divided into training and test sets containing 80% and 20% of the total cohort respectively; this division was stratified by overdose events after follow-up, so that the balance of target classes was preserved across the training and test sets.

Each model was trained using only the training set and all evaluation metrics were calculated from the model performance on the test set. To evaluate the performance of classifiers the metrics used are Accuracy, Area Under Receiver Operating Characteristic Curve (AUC), Sensitivity, and Specificity. We report AUC (also known as the C-statistic) as it is the standard—and sometimes only—metric to be reported. However, it is known for highly imbalanced datasets like this one, where the number of individuals in the dataset experiencing an overdose event after the index point is a very small proportion of those who do not, that AUC is sometimes misleading [43], in that a high AUC score is possible even where no overdose event is correctly predicted. Consequently, we also report sensitivity and specificity. In addition, each model was assessed at two different prediction thresholds of 0.5 and 0.1 to explore both balanced prediction and sensitivity-biased prediction.

Another approach to handling rare event prediction in imbalanced datasets is the use of risk stratification [13]. Rather than predicting a binary outcome for patients across the whole test set and evaluating how many of the predictions are correct, we identified a fixed proportion (10% in this case) of patients in the test set that have the highest predicted overdose risk score, then evaluated what proportion of the total minority outcomes (overdoses) are in this subset. This allows us to estimate what proportion of the total overdose events that could potentially be prevented subject to successful clinical intervention in the given proportion of highest-risk cases. For the evaluation of models, in addition to the standard accuracy, AUC, sensitivity and specificity, we report the proportion of total recorded overdoses in the test set within the top 10% of risk scores.

#### Interpretability

In order to understand how models are making predictions, we also report feature importance estimates. For logistic regression, the fitted coefficients represent the weight the model assigns to a feature. XGBoost also comes with an associated method for determining feature importance.

In addition, to investigate the PDC variable in particular, we use Partial Dependence Plots (PDP) [44] and Individual Conditional Expectation (ICE) plots [45]. PDPs represent the marginal effect of a feature on an outcome across the whole dataset, while ICE plots visualise the dependence of a feature on each instance; a PDP is thus the average of all ICE plots. This gives an insight into how the PDC variable varies with our OD outcome.

To quantify how much a change in PDC would affect an OD outcome, we used propensity score matching [46]. Specifically, we used the common nearest neighbour matching method, which allows us to pair individuals with an OD outcome with the closest individual who did not experience an OD outcome. This allows the calculation of an odds ratio to assess the effect of adjusting medication adherence.

As an additional sensitivity analysis, we consider effects on two predictive model if region-level variables are not included.

## Results

### Exploratory data analysis

After initial pre-processing and feature engineering, we summarised, visualised and explored the patient data to better understand the sample, underlying structures and relationships.

#### Cohort characteristics

A total of 26,685 patient records were identified with OUD diagnosis, OUD medication and appropriate Medicaid eligibility coverage. Age of the cohort is approximately normally distributed about a mean of 37.8 years old, with 56.49% of patients being female (n = 15,076), and 43.5% being male (n = 11,609). S1 Fig in the Supplementary Material shows the age distribution of the patients in the dataset.

#### Treatment medication

The most common treatment approach after the first diagnosis involves Buprenorphine prescription (75.97%) followed by prescriptions for both Buprenorphine and Naltrexone (14.1%) then Naltrexone alone (8.04%). Treatment involving Methadone is rare, with less than 1.9% of patients claiming a Methadone prescription in their first post-diagnosis period. S2 Fig shows the distribution.

#### Comorbidities

Depression was the most commonly occurring comorbid condition before diagnosis, affecting 24.31% of the cohort. Other conditions that commonly occur in general populations were also common in the cohort including hypertension (17.5%) and obesity (7.64%). Psychosis (13.5%), other neurological conditions (10.0%), and alcohol-related problems (8.5%) were unusually common in the cohort and potentially related to OUD. S3 Fig shows the distribution.

#### Outcome variable: Overdose events

As noted, the primary outcome of interest, an overdose event, was rare. In the cohort of 26,685, only 4,198 patients (15.7%) had a recorded overdose event. Only 2,753 patients (10.3%) had an overdose at any time after their first opioid-related diagnosis. 509 (1.9%) had an overdose in the 3-month treatment after the first diagnosis and 1,813 (6.79%) had an overdose in the 12 month observation period after the index point. The rarity of predictable overdose is expected but challenging for predictive models. S4 Fig shows the distribution.

Pearson correlation coefficients between overdose in the 3 months following diagnosis and all available predictor variables indicated mostly weak correlations, and the best potential predictor of an overdose in the prediction window was a prior overdose. Other neurological conditions and fluid/electrolyte problems were the most correlated comorbid conditions, although the relationship between these conditions and overdose is unclear. Claiming an ongoing opioid prescription was also correlated with overdose events in the prediction period. S5 Fig shows the 10 most highly correlated features.

#### Adherence to prescribed MOUD: PDC

For the 26,685 patients who claim an OUD treatment medication in the period following diagnosis, medication adherence scores (PDC) over the 3, 6 and 12 subsequent months were calculated. Visualizing the distribution of PDC for each period (S6 Fig), it is apparent that in the longer period (12 months) PDC is bimodally distributed with peaks at very high PDC (> 0.9) and very low PDC (< 0.1), indicating that patients tend to stick to their prescriptions or cease taking medication entirely, rather than continue with large gaps and partial coverage.

Over 7000 patients maintained PDC over 0.9 for 12 months or more: that is, a significant proportion of patients were committed to the very long-term treatment paradigms often used in MOUD.

Comparing PDC for each OUD medication type (S7 Fig) shows that PDC was significantly higher for Buprenorphine and Methadone over Naltrexone. It is unclear whether this was due to patient behaviour or differences in how the medications are prescribed, although we do note that patients generally preferred Buprenorphine/Naloxone combinations because they reduce cravings for opioids (especially short acting prescription opioids and heroin), while Naltrexone does not have that effect. As observed in S2 Fig, Naltrexone constituted only a small proportion of the medications, so perhaps not too much can be made of this difference.

Pearson correlation coefficients between medication adherence over 6 months (i.e. PDC > 0.8) and all available predictors were low (< 0.1 correlation). Previous and ongoing opioid prescriptions were associated with higher levels of adherence. This may be an indication that patients who develop OUD as a result of prescription medications rather than illicit drugs will have greater adherence with MOUD. Prior alcohol issues and prior overdose events, which would typically be indicators of more severe OUD, were also positively correlated with medication adherence. This could be a result of more serious problems corresponding to greater commitment to treatment, or evidence that less severe cases were not prescribed consistent long-term courses of Buprenorphine or Methadone. S8 Fig gives the top 10 correlated features. The table in S2 Appendix gives a description of the data broken down by medication adherence across all features.

Our key research question focuses on the association between PDC and OD events. We hypothesised that higher PDC was associated with lower risk of OD. S9 Fig shows the average OD rate for different ranges of PDC within the dataset. Although we cannot reach any definitive conclusions without considering the effect of the confounding variables, the overall trend of reduction of the OD rate with the increase of the PDC is as expected.

### Predictive modelling results

#### Overdose prediction at 12 months follow-up

Following are the results of all classifiers on the model, where the target variable is Overdose event in 12 months following the follow-up date. Table 2 summarizes results of all classifiers on the model. The XGB classifier had the highest AUC of 0.765, closely followed by the LR classifier (0.753). The LR classifier had the best stratification result: patients in the top 10% of risk scores account for 35.37% of overdose events over the next 12 months. While the Random Forest was also relatively high-performing, the Decision Tree model is poor: AUC is barely above chance, and stratification was close to what would be expected if risk scores were uniformly randomly distributed. Decision Trees are known to be low bias-high variance [47], and so may overfit to the training data, which appears to be the case here. Nevertheless, they form a useful constrast with the Random Forest learner, given that it is an aggregation of decision trees with the purpose of reducing the variance. These results are for all features: when applying backward stepwise elimination for feature selection, there was no essential improvement in model performance.

**Table 2:** Summary of results of all classifiers.

			Threshold (0.1)			Threshold (0.5)		
Model	Stratification (top 10%)	AUC	Accuracy	Sensitivity	Specificity	Accuracy	Sensitivity	Specificity
Logistic Regression	35.37	0.7530	0.8598	0.4147	0.8874	0.9415	0.0032	0.9996
Decision Tree	13.18	0.5216	0.8772	0.1189	0.9242	0.8787	0.1157	0.9260
Random Forest	28.61	0.7384	0.8724	0.2926	0.9082	0.9411	0.0064	0.9990
XGB	32.79	0.7656	0.8759	0.3601	0.9078	0.9417	0.0000	1.0

It is worth noting that the sensitivity of all models using a balanced (0.5) classification threshold was lower compared to the biased threshold (0.1), emphasising the importance of using a biased threshold or taking other steps to adapt to the highly skewed target class.

To visualize the numbers of patients this reflects, we give the confusion matrix for the LR classifier at threshold 0.1 in Table 3, the classifier with the largest number of true positives. It can be seen that with the class imbalance, the number of true negatives dominated, but relative to the other models it was relatively balanced in identifying positive cases, giving it the highest sensitivity.

**Table 3:** Confusion matrix for the logistic regression model, threshold = 0.1.

		Predicted	
		Negative	Positive
	Negative	4460	566
**Actual**	Positive	182	129

**Fig 2:**
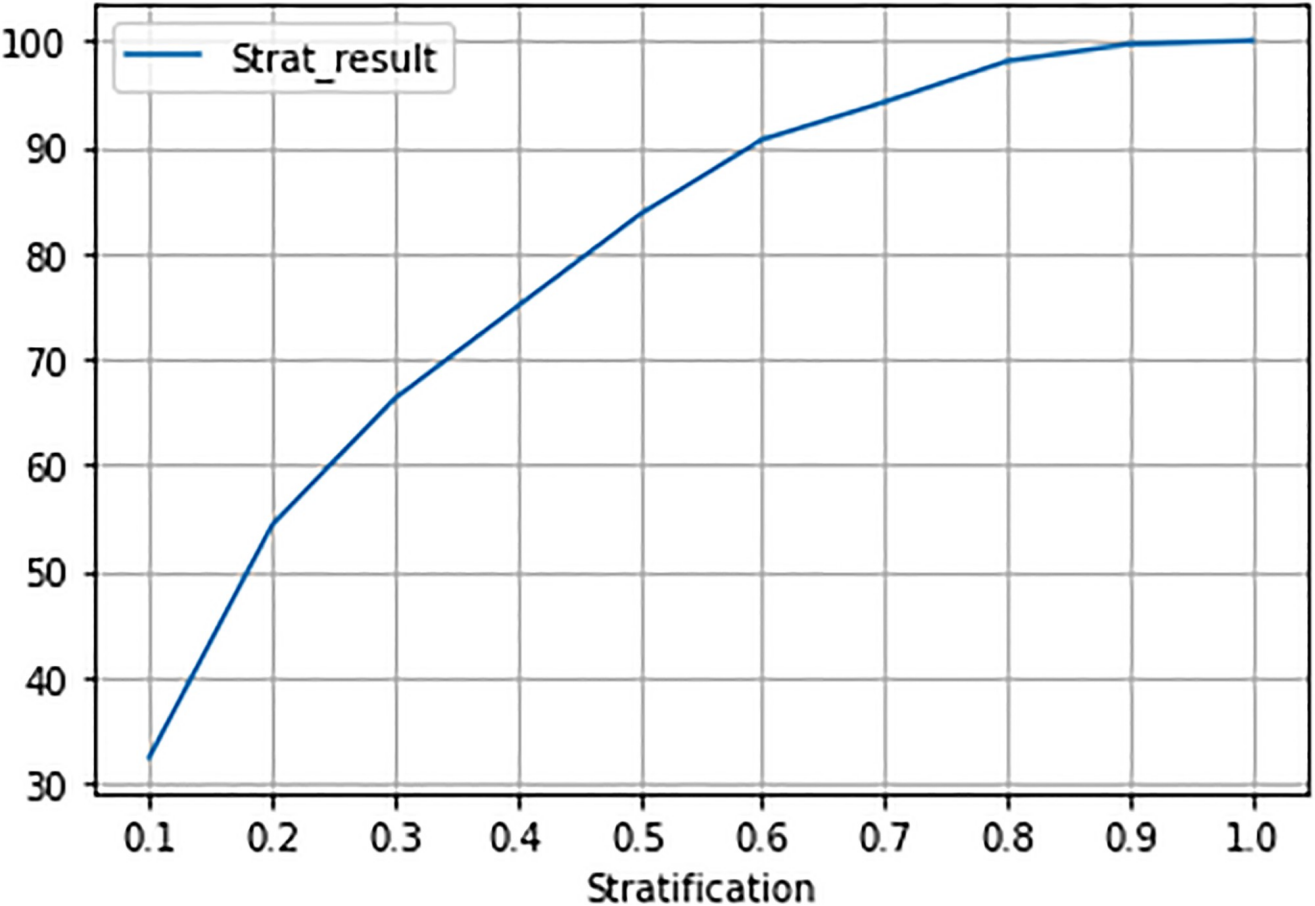
Risk stratification for XGB model.

The risk stratification graph for XGB (Fig 2), the classifier that performed best by AUC, shows the percentage of captured overdose events for different values of the risk strata. Each stratification decile contains 10% of the test set patients. The top 3 predicted risk deciles contain 66.2% of patients who experienced an overdose event in the 12 months after follow-up.

**Fig 3:**
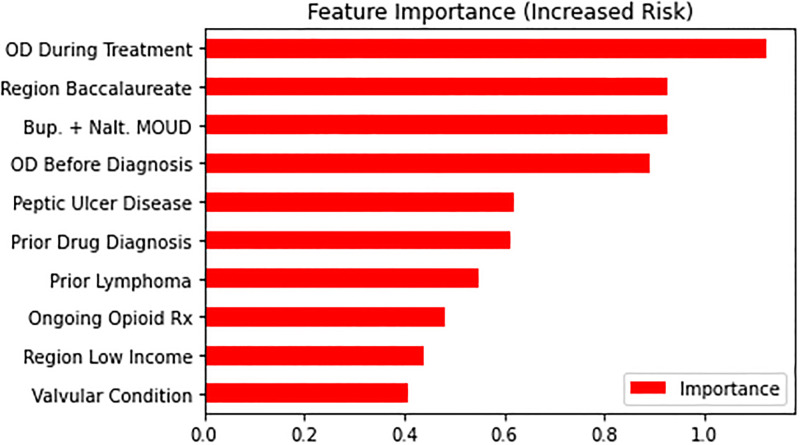
Top features with an increased risk of overdose (Logistic regression).

**Fig 4:**
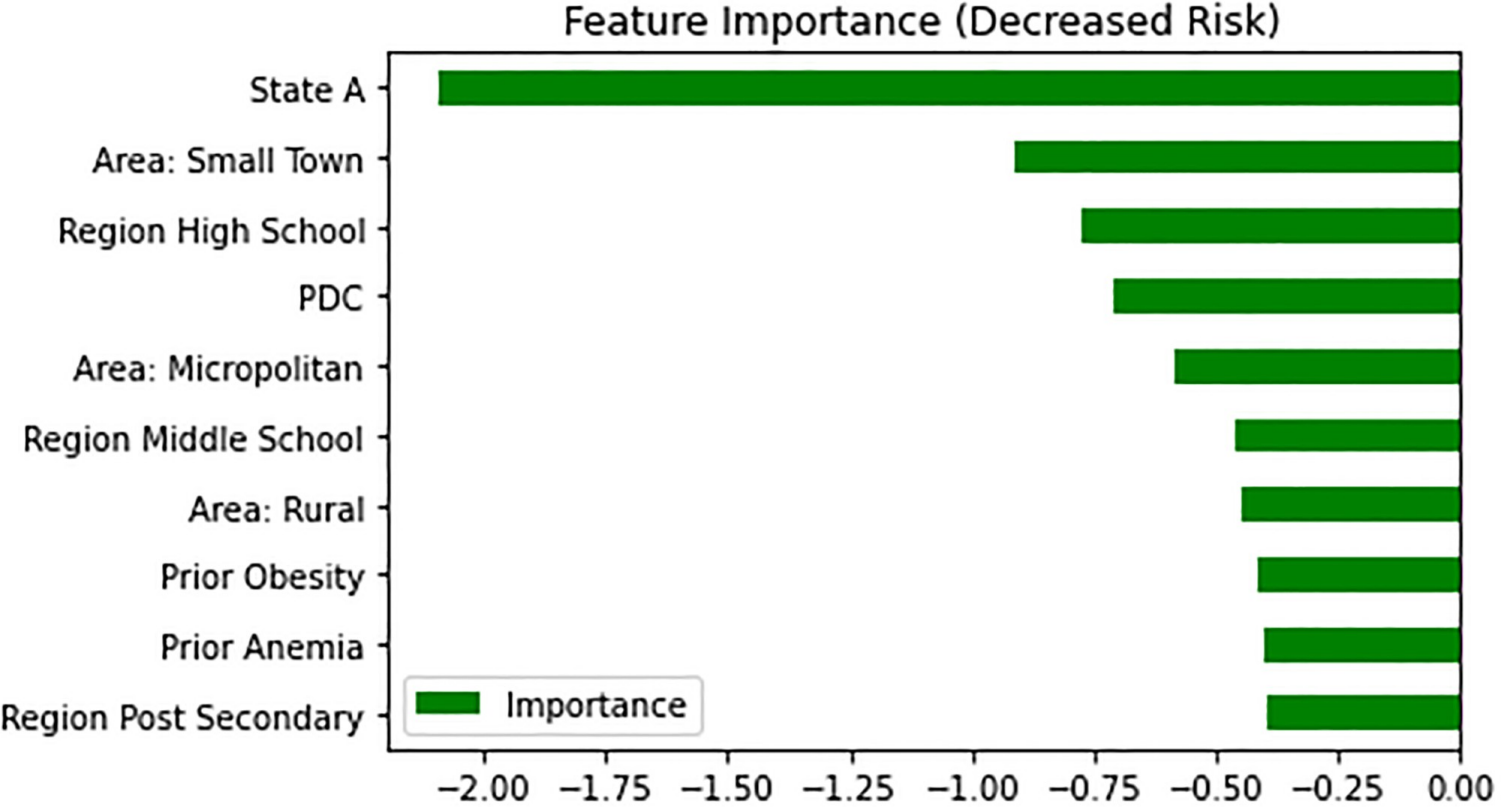
Top features with decreased risk of overdose.

We plotted estimated feature importance in predicting overdose events after follow-up using the LR classifier, as it is high performing and feature weights are easily interpretable under this model, highlighting the features that increase or decrease risk the most. For factors associated with higher risks (Fig 3), prior overdoses both before (od_before_f11) and after the first F11 diagnosis (3 month window, od_window_0) were important ones. Notably, in features associated with decreased risk of overdose (Fig 4), we found the PDC score calculated over the treatment window to be the among the most important predictors.

**Fig 5:**
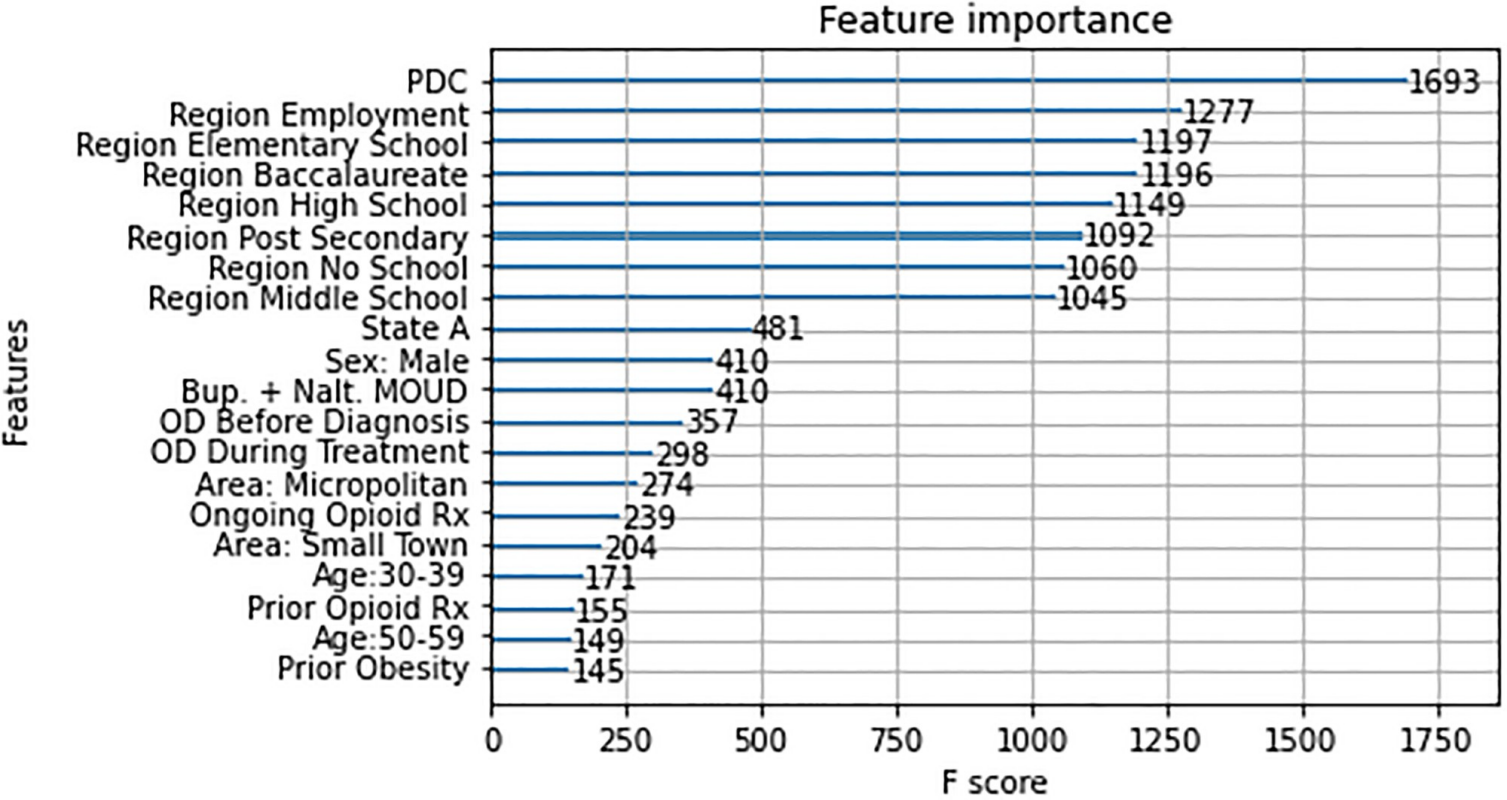
Feature importance for the XGB classifier.

For the XGB model, the feature importance method (Fig 5) reported that PDC score calculated over the treatment window was the most important feature of all, followed by employment and level of education (elementary_school, post_baccalaureate, post_secondary_edu, …).

**Effect of PDC: Removing the variable**. Another way of considering the value of PDC in the model is to compare our core model above, which contains PDC as a variable, with one that does not. In this variant model, everything was the same except for the absence of PDC as a variable. Table 4 summarises the comparison, and shows that all the high-performing models improved with PDC as a variable. The only one where it did not is the poorly performing Decision Tree, where results were still close to chance with or without PDC. (Given this at-chance performance, the marginally higher performance for Decision Tree without PDC is likely to be a random effect.)

**Table 4:** Summary of results.

	With PDC		Without PDC	
Model	Stratification (top 10%)	AUC	Stratification (top 10%)	AUC
Logistic Regression	35.37	0.7530	33.12	0.7325
Decision Tree	13.18	0.5216	16.39	0.5347
Random Forest	28.61	0.7384	28.61	0.7218
XGB	32.79	0.7656	31.83	0.7382

**Fig 6:**
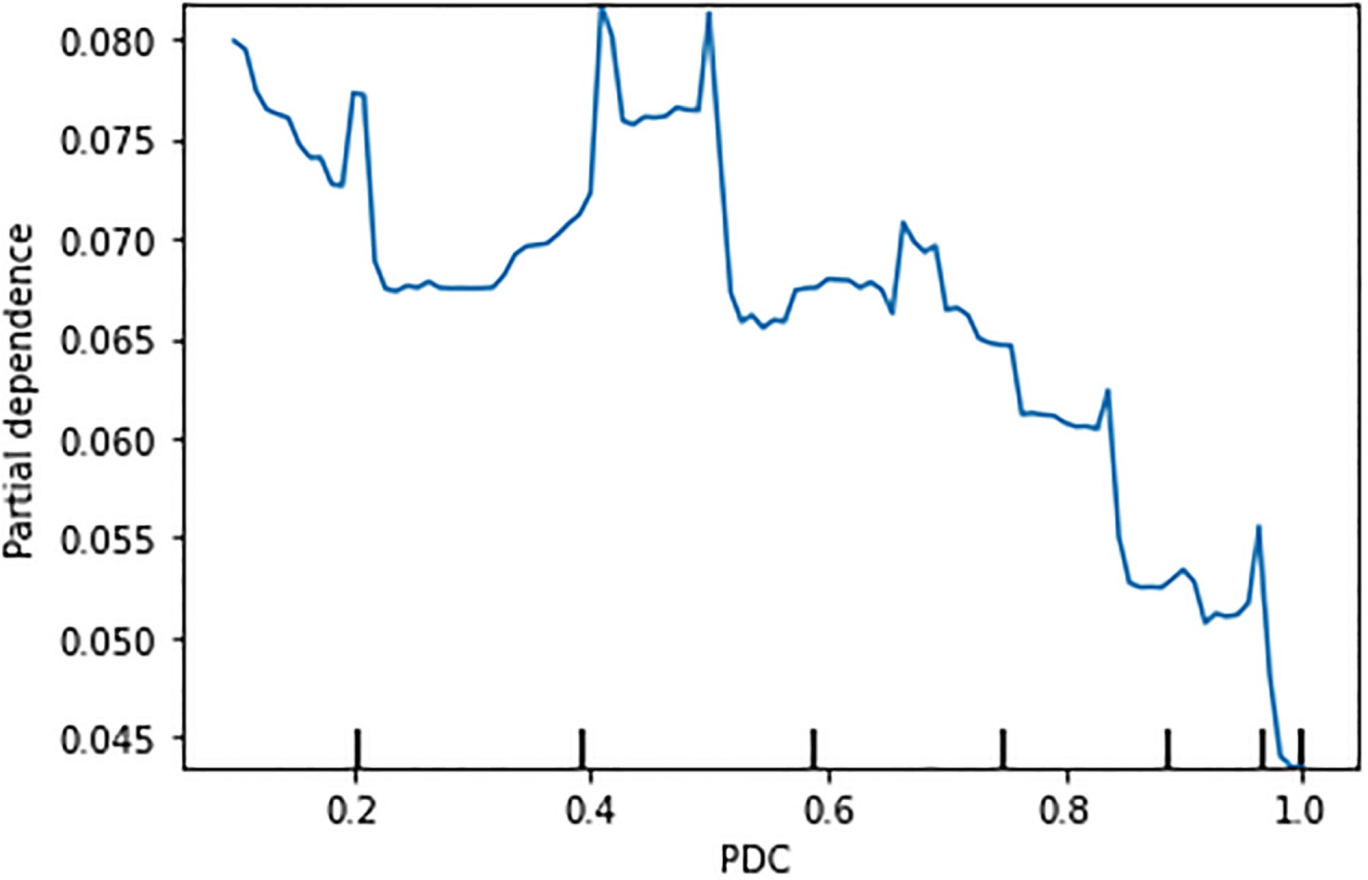
Partial dependence plot for the effect of PDP in XGB model.

**Fig 7:**
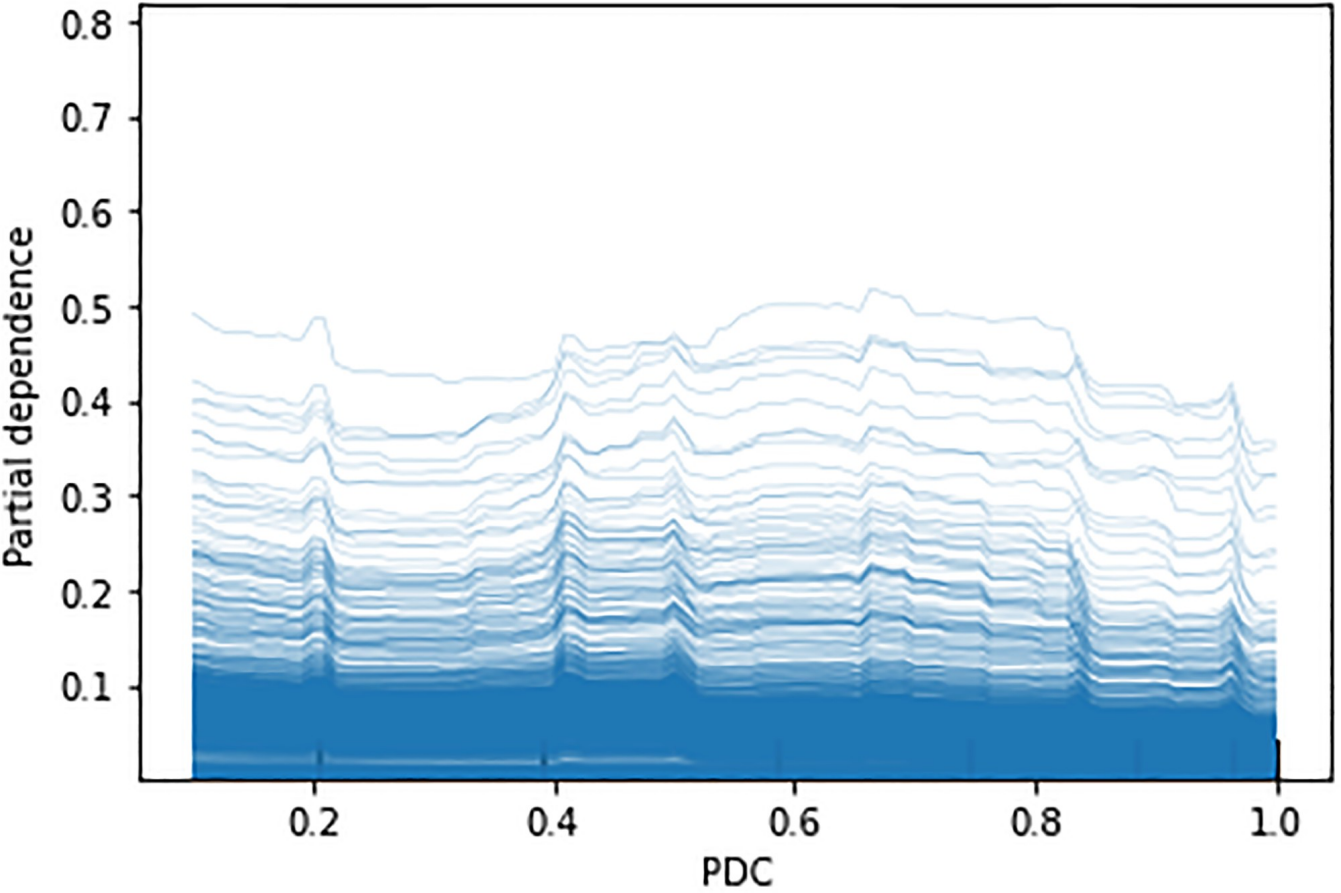
Individual conditional expectation (ICE) plot for the effect of PDP in XGB model.

**Behaviour of PDC: PDP and ICE plots**. Fig 6 shows an overall reduction of the OD rate with the increase of the PDC with some peaks in the middle. However, a particularly notable reduction happened when PDC crosses the 0.8 value. This is in line with the suggested threshold for the PDC value to define medication adherence in the literature as noted above. The ICE plot (Fig 7) shows that the same trend held for most of the individual instances in the data.

**Effect of medication adherence: Propensity score matching**. Inclusion of PDC as a predictor variable calculated over the 3 months between diagnosis and follow-up improved accuracy in long-term modelling. Both the logistic regression model and the XGB classifier indicated that high PDC values over the period were associated with a lower estimated risk of overdose, consistent with clinical literature surrounding MOUD. Further analysis on the effect size of adherence using a nearest neighbour matching method indicated that the cohort with medication adherence (PDC>0.8) have 59% less chance of having OD (OR = 0.41, 95% CI = (0.36, 0.46)).

The top 10 percent predicted scores in our best model could detect 35.3% (n = 112) of the people in the test set who had OD, of which 72.3% (n = 81) were not adhering to their medication. This means targeting that top 10 percent predicted cohort with an intervention encouraging them to adhere to their medication could be beneficial for 25.5% (35.3%×72.3%=25.5%) of all cases with OD. Considering the calculated effect of medication adherence in our matched population (OR = 0.41, 95% CI = (0.36, 0.46)), on average, such intervention may result in 10.4% fewer cases of OD in the population (0.41×0.255% =10.4%).

**Effects of removing region-level variables**. Since our limited dataset lacks the patient’s socio-economic status, we generated some proxy features by leveraging the patients’ Zip codes and supplemental region-level data. As noted above, some of these features showed up among the important features of our models. To investigate the effectiveness of these added features, and also to see whether their inclusion obscures the effects of other individual features, we excluded them (income, education and employment) from the models and compared the results with the previous models. Excluding these features decreased both accuracy and the stratification results for both LR and XGB models. AUC remained the same for LR but decreased in the XGB model. Removing these features made room for other features to appear among the top 10 positive and negative coefficients in the LR model. Prior diabetes mellitus (Prior_DM) and Age category 50 to 59 (age_cat_50_59) moved into the top 10 features decreasing the risk of the future OD. Prior Metastatic cancer (Prior_Mets) and prior weight loss (Prior_WeightLoss) joined the top 10 factors increasing the risk. The values of the coefficients for the Urban development features also increased. Having prior obesity among the factors that decrease the chance of OD and prior weight loss among those that increases the chance may seem unexpected. Although many studies suggest that there is an association between obesity and chronic opioid prescription and use through the association of excess weight and chronic pain [48, 49], a 2018 study [50] reveals an inverse relationship between obesity prevalence and opioid dependence, and [51] characterises weight loss as an alert for substance use problems which seems in line with our findings.

## Discussion

Given the seriousness of opioid overdose, any ability to identify high-risk individuals and provide preventative intervention is of great value to medical practitioners and patients. While doctors have traditionally manually made assessments of risk, based on their professional expertise and the presentation of the patient, there is an abundance of additional data and complicated interactions that may be relevant to predicting overdose. The evaluation metrics and risk stratification results achieved by the model clearly show that predicting overdoses using machine learning techniques is possible and worth developing further. While the sensitivity and positive predictive values are low compared to some classification tasks, the risk stratification results are potentially useful in the context of potential clinical interventions. All models in the project operate on de-identified claims level data, requiring less detailed personal and medical information, reducing (but not eliminating) the privacy concerns of using such a model in the real world. The models also provide the reported accuracies using only medical history claimed under Medicaid with a 3-month prior eligibility minimum, rather than complete and comprehensive patient histories.

### Rare outcome prediction

The prediction of rare events like opioid overdose using machine learning is a challenging issue. The percentage of observations with a positive outcome is quite small in the dataset, approximately 7%. This makes it very challenging for machine learning algorithms to predict the positive outcomes consistently. One possible approach to mitigating the effect of this sparsity on predictive modelling is to use techniques to rebalance the training dataset, such as for example SMOTE [52], which generates synthetic training data and which often improves model performance across metrics. In this case, this would involve synthetically increasing the proportion of patients with overdose events (minority class) upwards from 7%. We carried out some preliminary efforts in using SMOTE, but did not find any positive outcomes: [52] note the challenges of binary and categorial predictors, even for variants of SMOTE that are designed for these sorts of variables. A deeper investigation on training set rebalancing may, however, be more fruitful.

Given the existing dataset imbalance, in this paper we have presented models that make probabilistic predictions of overdose risk, which can be used to stratify patients and identify high-risk groups. It is unlikely that very high sensitivity scores will be feasible in this kind of rare event prediction; however, risk stratification approaches can identify significant proportions of those patients who will experience adverse outcomes and assist in directing resources and attention to the patients who are most at risk. Models that support this kind of output have several advantages over hard classifiers in this domain, making them a sensible focus of future research. They can improve the transparency and interpretability of systems by providing understandable risk groups to support classifications as well as allow practitioners and policymakers to explore the potential impact of intervention at different thresholds.

### Limitations and potential improvements

The highest performing models obtained an AUC of 0.76, which has significant room for improvement. The predictive accuracy of all models could improve given more complete or more detailed background information. There is a balance to be struck between the accuracy of predictive models and limiting the applicability of models by requiring new data sources. Therefore, extracting the maximum value and detail from the available claims data should be the highest priority for future work in this area.

The current work involved compressing large scale temporal data, i.e. claims events occurring on a timeline, into simple features suitable for a range of machine learning algorithms. Part of this process involved encoding previous diagnoses into categorical prior comorbidity categories as outlined in the feature engineering section. While this approach dramatically reduces the number of features to consider and is a simple, reproducible process using publicly available libraries, it does not differentiate specific conditions within those that may be relevant to OUD and overdose prediction. For example, the “Prior Lymphoma” comorbidity category is a strong predictor of overdose risk in the model. This category includes at least one condition that is associated with overdose risk; however, it is very unlikely that all conditions in this category are associated with an increase in risk. Therefore, building comorbidity categories through feature engineering that are specific to the task of identifying overdose risk could improve the quality of this type of predictor and the predictive power of the model.

Similar to the pre-processing used for comorbidities, medication history is summarised into a limited number of binary variables representing broad drug categories. Consideration of medications individually rather than by category and including dosage and prescription duration over the patient history could improve the predictive power of models. This kind of dosage consideration was found to be useful in previous studies that examined the risk of developing OUD particularly concerning opioid dosages. While including more detailed features derived from medical and prescription history increases the complexity of required models, modern machine learning techniques can operate well over very large numbers of heterogeneous predictors and since this information is available in the claims data, it should certainly be considered in future work. The examination of medications in this work is also limited by the fact that this type of data contains a history of claims, rather than an authoritative history of use. Medications, in some cases, could have been acquired and claimed for but not used, or used incorrectly. The use of claims history also does not allow differentiation between low levels of medication adherence and cessation of medication for other reasons, including the direction of treating physicians. This means that features representing medication history as well as PDC scores used to examine medication compliance are imperfect measures of true medication usage.

Secondary to better use of the primary data source, additional sources of data including more detailed external demographic and geographic information could be considered. One notable limitation of the claims data is that overdose events resulting in death without hospital admission are not present in the claims data. This lack of mortality data is a problem for the current work since the worst possible outcomes may appear to be positive outcomes (i.e. no recorded overdose event). Incorporation of this data would be of great value, if possible. The successful use of region-level data in this work (several region-level variables were high in feature importance estimates) implies that this method of joining has merit, opening up a wide range of demographic, economic and geographical data that could be included as features in future work. It must be noted, however, that while these region-level variables are useful in improving predictive accuracy, they are not independent of each other at the level of the individual patient. This limits the strength of inferences about any individual region-level variable.

An additional area for future work is to explore a wider range of models. We looked at several not reported here: Support Vector Machines (SVMs), for example, take a long time to converge to a solution and do not score better on any metrics; a fully connected Deep Neural Network similarly did not perform better than the models reported. However, there are more advanced types of neural networks that can be applied to temporal data that could show better performance in this context.

## Conclusion

In the past decade, the opioid crisis in the United States has gotten increasingly worse. This poses a serious concern for the American public. Patients diagnosed with substance use disorders are often people with social problems, as well as physical and psychological comorbidities. Some of these factors are not easily changeable, but improvement in medication adherence is one that can potentially lead to improved outcomes.

To investigate this, we built predictive machine learning models that included medication adherence as an independent variable, using Medicaid datasets from two US states. Our models had good predictive power, with the XGB classifier the best overall (AUC 0.7656). Risk stratification results showed that using the classifiers to identify the 10% most at-risk individuals can capture up to 35.37% of all overdose cases, making this a potentially useful tool in clinical interventions. Adherence to medication was one of the most important variables in the models, and an analysis supported the conventional 80% threshold for deeming individuals adherent. Given this threshold, our models suggest that improving medication adherence among non-adherent individuals in the most at-risk categories could reduce overdose outcomes by around a quarter in that group, and in around 10% of the overall cohort.

This model offers significant advantages for patient outreach to those who are at risk for opioid overdose. A primary obstacle to outreach is the high cost due to cost of professional outreach resources. By narrowing down the risk group it allows more effective use of outreach resources to focus on those more at risk. It also highlights that a key area of emphasis for outreach should be medication adherence. The model shows that this should be a primary feature of outreach activities. Giving this kind of guidance allows care managers and others doing patient outreach to be more effective and efficient by making sure to focus their efforts in an area that has been shown to make a difference limiting adverse outcomes.

Future work would include investigating how to address various limitations in order to improve the predictive accuracy of the models. In particular, this could involve using a finer granularity of the claims data, including looking at comorbidity categories and temporal aspects of claims in ways specific to this problem.

## Supporting information

S1 Appendix Predictor feature details. (PDF)

S2 Appendix Description of the data broken down by medication adherence. (PDF)

S3 Appendix Hyperparameters used in the models. (PDF)

S1 Fig Age distribution of the cohort. (PNG)

S2 Fig Frequency of medications in the cohort. (PNG)

S3 Fig Frequency of top 10 comorbidities present in the cohort. (PNG)

S4 Fig Frequency of patients who have overdoses by time period, after they join Medicaid. (PNG)

S5 Fig Top 10 features in order of decreasing correlation with overdose, within 3 months. (PNG)

S6 Fig Distributions of PDC and Adherence over 3-months, 6-months, and 12-months periods, respectively. (PNG)

S7 Fig Distribution of PDC based on medication type. (PNG)

S8 Fig Top 10 features that are decreasingly correlated with Adherence. (PNG)

S9 Fig Average OD rate for cohorts with different PDC (12 months). (PNG)

S10 Fig Top features with an increased risk of overdose after excluding socio-economic features (Logistic regression). (PNG)

S11 Fig Top features with decreased risk of overdose after excluding socio-economic features. (PNG)

S12 Fig Feature importance for the XGB classifier after excluding socio-economic features. (PNG)

S1 File (PDF)
