# Supplementary material for: Using machine learning to study the effect of medication adherence in Opioid Use Disorder
Source: PLoS One. 2022 Dec 15;17(12):e0278988. doi: 10.1371/journal.pone.0278988 (PMC9754174; doi:10.1371/journal.pone.0278988)
Supplement: S1 Appendix — (PDF) [file pone.0278988.s001.pdf]

S1 Appendix: Predictor Feature Details

Calculation of Proportion of Days Covered (PDC) PDC is calculated by establishing the number of days a patient could have their medication given the history of prescription claims and supplied amounts, then dividing this by the number of days in the period of interest. To account for the different prescription patterns that may be used in different medication types, we use the "days supply" indicated at each prescription claim, allowing us to estimate the PDC independent of the prescription pattern (i.e. daily, weekly, monthly refills).

$$\text{PDC} = \frac{\text{Days Covered}}{\text{Total Days in Period}}$$

where days covered is calculated by:

1. Identifying all prescription claims and days supply for MOUD in the examined period;
2. Iterating through prescriptions in chronological order counting any "gap" days where the patient is not covered and adding any overlapping days to the next claim; and then
3. Deriving days covered by subtracting the total gap days from the days in the period.

Treatment medication An important factor in determining outcomes is the treatment approach chosen by the treating doctor including both whether prescription medication is used and which type of medication is prescribed for the OUD. Pharmacy claims in the first 3 months after the first diagnosis are scanned for any of the primary treatment medications. The medication with the largest number of claims in the period is considered to be the MOUD approach prescribed at the diagnosis point. A special case is considered where both Buprenorphine and Naltrexone are claimed in the treatment period in order to capture protocols combining the medications [1, 2]. This treatment approach is then encoded into a categorical predictor variable for all models indicating window medication as one of:

- Buprenorphine
- Methadone
- Naltrexone
- Buprenorphine and Naltrexone

Comorbidities The 29 categories for comorbidity are: Congestive Heart Failure (CHF), Valvular heart problems, Pulmonary Hypertension (PHTN), Peripheral vascular disease (PVD), Hypertension (HTN), Paralysis, Other Neurological (NeuroOther), Pulmonary, Diabetes (DM), Diabetes with complications (DMcx), Hypothyroid, Renal issues, Liver issues, Peptic Ulcers (PUD), HIV, Lymphoma, Metabolic disorders (Mets), Tumour, Rheumatic issues, Coagulopathy, Obesity, Weight Loss, Electrolyte problems (FluidsLytes), Blood Loss, Anaemia, Alcohol issues, Drug-related problems, Psychosis and Depression.

The targeted categories are then simplified into 29 binary variables. Each one indicating the presence of at least one diagnosed comorbid condition in the given category before OUD diagnosis. These binary variables represent a simplified medical history that can easily and realistically be used in predictive modelling at the time of first OUD diagnosis. This approach has many advantages:

1. Claims-level data without indicators of severity can be used.
2. It is simple and reproducible given a history of diagnosis codes.
3. Complex patient history can be considered without overwhelming the model with trivial features.
4. Features have a clear and interpretable general clinical meaning improving interpretability.

Other prescriptions NDC codes for drugs of the classes of drugs included as predictors (SSRIs, benzodiazepine class drugs, opioid based analgesics) are extracted from the RxNorm API via the RxNormR R package then used to scan prescription claims for the cohort. Prescriptions from the 3 classes are partitioned into:

- "Prior" - claimed before the patient's first OUD diagnosis.
- "During" - claimed during the month following the first OUD diagnosis.

This separation is made to differentiate historical factors from potential pharmaceutical interactions in co-prescribed medications. The presence or absence of a prescription claim in each class for each partition is then encoded as a binary variable (6 total). An assumption is made that some ongoing prescriptions a patient will use during treatment for OUD can be determined at the point of diagnosis.

The complete lists of drugs in each class follows.

List of SSRIs

- Citalopram (Celexa)

- Escitalopram (Lexapro)
- Fluoxetine (Prozac)
- Paroxetine (Paxil, Pexeva)
- Sertraline (Zoloft)
- Vilazodone
- Fluvoxamine

List of BENZODIAZEPINES

- Alprazolam (Xanax)
- Chlordiazepoxide (Librium)
- Diazepam (Valium)
- Lorazepam (Ativan)
- Triazolam
- Estazolam
- Temazepam
- Quazepam
- Flurazepam

List of Opioids

- Opium
- Heroin
- Codeine
- Oxycodone
- Hydrocodone
- Tramadol
- Morphine
- Hydromorphone
- Fentanyl

- Carfentanil
- Vicodin
- Percocet

Income Household income is included as a single categorical variable by dividing regions into income categories based on average household income quantiles:

- Low Income (quantile < 0.2)
- Medium Income (quantile between 0.2 and 0.8)
- High Income (quantile > 0.8)

Source - Household Income in the Past 12 Months (In 2018 Inflation-adjusted Dollars) American Community Survey 5-year estimates (2014-2018) from the US Census Bureau.

Education Education factors are represented as six numeric variables representing estimated proportions of the adult population with the highest level of educational attainment given by:

- No School
- Elementary School
- Middle School
- High School
- Post-Secondary Education
- Post-Baccalaureate Education

Source: Educational Attainment for the Population 25 Years and Over American Community Survey 5-year estimates (2014-2018) from the US Census Bureau.

Employment Employment is represented by three numeric variables indicating the proportion of the population in the area:

- Unemployed (looking for work)
- Employed
- Not in Labour Force

Source: Employment Status for the Population 16 Years and Over American Community Survey 5-year estimates (2014-2018) from the US Census Bureau.

Urban development Urban development is indicated by a single categorical variable indicating whether an area is considered urban or rural according to its level of development. Source: Economic Research Service (ERS) of the United States Department of Agriculture. Rural-Urban Commuting Area Codes.

References

1. Gerra G, Fantoma A, Zaimovic A. Naltrexone and buprenorphine combination in the treatment of opioid dependence. J Psychopharmacol. 2006 Nov;20(6):806-14. doi: 10.1177/0269881106060835.

2. Sigmon SC, Bisaga A, Nunes EV, O'Connor PG, Kosten T, Woody G. Opioid Detoxification and Naltrexone Induction Strategies: Recommendations for Clinical Practice. Am J Drug Alcohol Abuse. 2012;38(3):187-199. doi: 10.3109/00952990.2011.653426.
