# Supplementary material for: Using machine learning to study the effect of medication adherence in Opioid Use Disorder
Source: PLoS One. 2022 Dec 15;17(12):e0278988. doi: 10.1371/journal.pone.0278988 (PMC9754174; doi:10.1371/journal.pone.0278988)
Supplement: S2 Appendix — (PDF) [file pone.0278988.s002.pdf]

S2 Appendix: Description of the data broken down by medication adherence

		Missing	Overall	PDC < 0.8	PDC ≥ 0.8	P-Value
n			26685	11564	15121	
indv_GENCD_RF, n (%)	F	0	15076 (56.5)	6322 (54.7)	8754 (57.9)	<0.001
	M		11609 (43.5)	5242 (45.3)	6367 (42.1)	
age_cat, n (%)	16-29	0	5240 (19.6)	2495 (21.6)	2745 (18.2)	<0.001
	30-39		11774 (44.1)	5069 (43.8)	6705 (44.3)	
	40-49		6031 (22.6)	2527 (21.9)	3504 (23.2)	
	50-59		2935 (11.0)	1226 (10.6)	1709 (11.3)	
	above_60		705 (2.6)	247 (2.1)	458 (3.0)	
Prior_CHF, n (%)	False	0	26370 (98.8)	11426 (98.8)	14944 (98.8)	0.909
	True		315 (1.2)	138 (1.2)	177 (1.2)	
Prior_Valvular, n (%)	False	0	26203 (98.2)	11387 (98.5)	14816 (98.0)	0.004
	True		482 (1.8)	177 (1.5)	305 (2.0)	
Prior_PHTN, n (%)	False	0	26505 (99.3)	11487 (99.3)	15018 (99.3)	0.939
	True		180 (0.7)	77 (0.7)	103 (0.7)	
Prior_PVD, n (%)	False	0	26239 (98.3)	11402 (98.6)	14837 (98.1)	0.003
	True		446 (1.7)	162 (1.4)	284 (1.9)	
Prior_HTN, n (%)	False	0	22435 (84.1)	9906 (85.7)	12529 (82.9)	<0.001
	True		4250 (15.9)	1658 (14.3)	2592 (17.1)	
Prior_Paralysis, n (%)	False	0	26573 (99.6)	11515 (99.6)	15058 (99.6)	0.995
	True		112 (0.4)	49 (0.4)	63 (0.4)	
Prior_NeuroOther, n (%)	False	0	24501 (91.8)	10629 (91.9)	13872 (91.7)	0.622
	True		2184 (8.2)	935 (8.1)	1249 (8.3)	
Prior_Pulmonary, n (%)	False	0	22288 (83.5)	9858 (85.2)	12430 (82.2)	<0.001
	True		4397 (16.5)	1706 (14.8)	2691 (17.8)	
Prior_DM, n (%)	False	0	25546 (95.7)	11110 (96.1)	14436 (95.5)	0.017
	True		1139 (4.3)	454 (3.9)	685 (4.5)	
Prior_DMcx, n (%)	False	0	25935 (97.2)	11258 (97.4)	14677 (97.1)	0.166
	True		750 (2.8)	306 (2.6)	444 (2.9)	
Prior_Hypothyroid, n (%)	False	0	25772 (96.6)	11231 (97.1)	14541 (96.2)	<0.001
	True		913 (3.4)	333 (2.9)	580 (3.8)	
Prior_Renal, n (%)	False	0	26471 (99.2)	11479 (99.3)	14992 (99.1)	0.316
	True		214 (0.8)	85 (0.7)	129 (0.9)	
Prior_Liver, n (%)	False	0	24380 (91.4)	10637 (92.0)	13743 (90.9)	0.002
	True		2305 (8.6)	927 (8.0)	1378 (9.1)	
Prior_PUD, n (%)	False	0	26502 (99.3)	11493 (99.4)	15009 (99.3)	0.243
	True		183 (0.7)	71 (0.6)	112 (0.7)	
Prior_HIV, n (%)	False	0	26570 (99.6)	11523 (99.6)	15047 (99.5)	0.116
	True		115 (0.4)	41 (0.4)	74 (0.5)	
Prior_Lymphoma, n (%)	False	0	26656 (99.9)	11553 (99.9)	15103 (99.9)	0.689
	True		29 (0.1)	11 (0.1)	18 (0.1)	
Prior_Mets, n (%)	False	0	26636 (99.8)	11539 (99.8)	15097 (99.8)	0.346
	True		49 (0.2)	25 (0.2)	24 (0.2)	

Prior_Tumor, n (%)	False	0	26437 (99.1)	11463 (99.1)	14974 (99.0)	0.442
	True		248 (0.9)	101 (0.9)	147 (1.0)	
Prior_Rheumatic, n (%)	False	0	25925 (97.2)	11247 (97.3)	14678 (97.1)	0.379
	True		760 (2.8)	317 (2.7)	443 (2.9)	
Prior_Coagulopathy, n (%)	False	0	26325 (98.7)	11424 (98.8)	14901 (98.5)	0.097
	True		360 (1.3)	140 (1.2)	220 (1.5)	
Prior_Obesity, n (%)	False	0	24766 (92.8)	10855 (93.9)	13911 (92.0)	<0.001
	True		1919 (7.2)	709 (6.1)	1210 (8.0)	
Prior_WeightLoss, n (%)	False	0	25934 (97.2)	11250 (97.3)	14684 (97.1)	0.414
	True		751 (2.8)	314 (2.7)	437 (2.9)	
Prior_FluidsLytes, n (%)	False	0	24875 (93.2)	10720 (92.7)	14155 (93.6)	0.004
	True		1810 (6.8)	844 (7.3)	966 (6.4)	
Prior_BloodLoss, n (%)	False	0	26457 (99.1)	11464 (99.1)	14993 (99.2)	0.926
	True		228 (0.9)	100 (0.9)	128 (0.8)	
Prior_Anemia, n (%)	False	0	25577 (95.8)	11112 (96.1)	14465 (95.7)	0.087
	True		1108 (4.2)	452 (3.9)	656 (4.3)	
Prior_Alcohol, n (%)	False	0	24785 (92.9)	10640 (92.0)	14145 (93.5)	<0.001
	True		1900 (7.1)	924 (8.0)	976 (6.5)	
Prior_Drugs, n (%)	False	0	32 (0.1)	10 (0.1)	22 (0.1)	0.229
	True		26653 (99.9)	11554 (99.9)	15099 (99.9)	
Prior_Psychoses, n (%)	False	0	23631 (88.6)	10228 (88.4)	13403 (88.6)	0.640
	True		3054 (11.4)	1336 (11.6)	1718 (11.4)	
Prior_Depression, n (%)	False	0	20774 (77.8)	9022 (78.0)	11752 (77.7)	0.571
	True		5911 (22.2)	2542 (22.0)	3369 (22.3)	
income_cat, n (%)	high	373	4017 (15.3)	1754 (15.4)	2263 (15.2)	<0.001
	low		4353 (16.5)	2022 (17.7)	2331 (15.6)	
	mid		17942 (68.2)	7630 (66.9)	10312 (69.2)	
no_school, mean (SD)		277	0.0 (0.0)	0.0 (0.0)	0.0 (0.0)	0.817
elementary_school, mean (SD)		277	0.0 (0.0)	0.0 (0.0)	0.0 (0.0)	0.422
middle_school, mean (SD)		277	0.0 (0.0)	0.0 (0.0)	0.0 (0.0)	0.108
high_school, mean (SD)		277	0.1 (0.0)	0.1 (0.0)	0.1 (0.0)	0.050
post_secondary_edu, mean (SD)		277	0.8 (0.1)	0.8 (0.1)	0.8 (0.1)	<0.001
post_baccalaureate, mean (SD)		277	0.1 (0.1)	0.1 (0.1)	0.1 (0.0)	0.003
employed, mean (SD)		264	0.5 (0.1)	0.5 (0.1)	0.5 (0.1)	0.336
area_type, n (%)	Metropolitan are	12	17894 (67.1)	8035 (69.5)	9859 (65.2)	<0.001
	Micropolitan area		6374 (23.9)	2545 (22.0)	3829 (25.3)	
	Rural areas		584 (2.2)	234 (2.0)	350 (2.3)	
	Small town		1821 (6.8)	742 (6.4)	1079 (7.1)	

window_mat, n (%)	B	0	22543 (84.5)	9470 (81.9)	13073 (86.5)	<0.001
	BN		1681 (6.3)	926 (8.0)	755 (5.0)	
	M		309 (1.2)	126 (1.1)	183 (1.2)	
	N		2152 (8.1)	1042 (9.0)	1110 (7.3)	
ssri_rx_before_f11, n (%)	False	0	18099 (67.8)	7920 (68.5)	10179 (67.3)	0.044
	True		8586 (32.2)	3644 (31.5)	4942 (32.7)	
ssri_rx_during_f11, n (%)	False	0	22177 (83.1)	9733 (84.2)	12444 (82.3)	<0.001
	True		4508 (16.9)	1831 (15.8)	2677 (17.7)	
bzd_rx_before_f11, n (%)	False	0	24725 (92.7)	10752 (93.0)	13973 (92.4)	0.081
	True		1960 (7.3)	812 (7.0)	1148 (7.6)	
bzd_rx_during_f11, n (%)	False	0	26231 (98.3)	11341 (98.1)	14890 (98.5)	0.014
	True		454 (1.7)	223 (1.9)	231 (1.5)	
od_before_f11, n (%)	False	0	25240 (94.6)	10839 (93.7)	14401 (95.2)	<0.001
	True		1445 (5.4)	725 (6.3)	720 (4.8)	
opd_rx_before_f11, n (%)	False	0	16885 (63.3)	7185 (62.1)	9700 (64.1)	0.001
	True		9800 (36.7)	4379 (37.9)	5421 (35.9)	
opd_rx_during_f11, n (%)	False	0	25236 (94.6)	10730 (92.8)	14506 (95.9)	<0.001
	True		1449 (5.4)	834 (7.2)	615 (4.1)	
od_window_0, n (%)	False	0	26087 (97.8)	11204 (96.9)	14883 (98.4)	<0.001
	True		598 (2.2)	360 (3.1)	238 (1.6)	
state, n (%)	State B	0	22315 (83.6)	9565 (82.7)	12750 (84.3)	<0.001
	State A		4370 (16.4)	1999 (17.3)	2371 (15.7)	
od_after_3m, n (%)	False	0	25132 (94.2)	10662 (92.2)	14470 (95.7)	<0.001
	True		1553 (5.8)	902 (7.8)	651 (4.3)	
od_after_F11, n (%)	False	0	24684 (92.5)	10393 (89.9)	14291 (94.5)	<0.001
	True		2001 (7.5)	1171 (10.1)	830 (5.5)	
top_pdc_3m, mean (SD)		0	0.7 (0.3)	0.4 (0.2)	1.0 (0.1)	<0.001
