# Supplementary material for: Using machine learning to study the effect of medication adherence in Opioid Use Disorder
Source: PLoS One. 2022 Dec 15;17(12):e0278988. doi: 10.1371/journal.pone.0278988 (PMC9754174; doi:10.1371/journal.pone.0278988)
Supplement: S3 Appendix — (PDF) [file pone.0278988.s003.pdf]

# S3 Appendix: Hyperparameters used in the models

| Model | Parameters |
|---|---|
| Logistic Regression | C = 1.0 |
| Decision Tree | criterion=gini, min_samples_split =2, min_samples_leaf =1 |
| Random Forest | n_estimators=200, criterion=gini, max_depth=10, random_state=0, max_features=None |
| XGB | learning_rate=0.01, n_estimators=1000, max_depth=6, gamma=10, subsample = 0.8, colsample_bytree = 0.4, scale_pos_weight=1, objective=binary:logistic, reg_alpha = 0.3 |
