# Supplementary figures and images for: Using machine learning to study the effect of medication adherence in Opioid Use Disorder
Source: PLoS One. 2022 Dec 15;17(12):e0278988. doi: 10.1371/journal.pone.0278988 (PMC9754174; doi:10.1371/journal.pone.0278988)

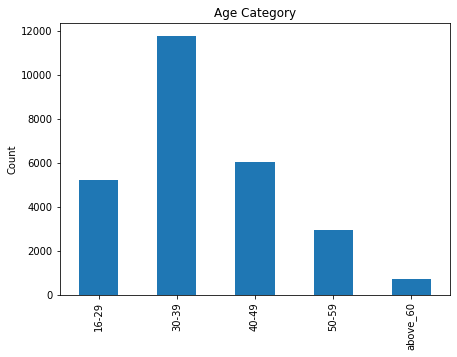

Supplement: S1 Fig — (PNG) [file pone.0278988.s004.png]

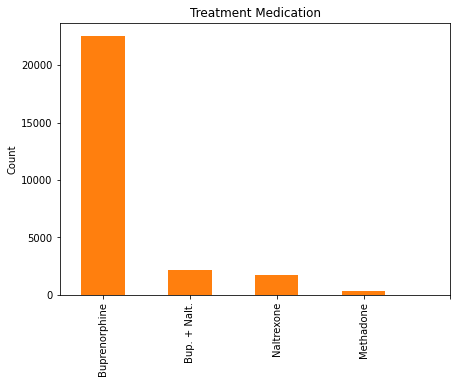

Supplement: S2 Fig — (PNG) [file pone.0278988.s005.png]

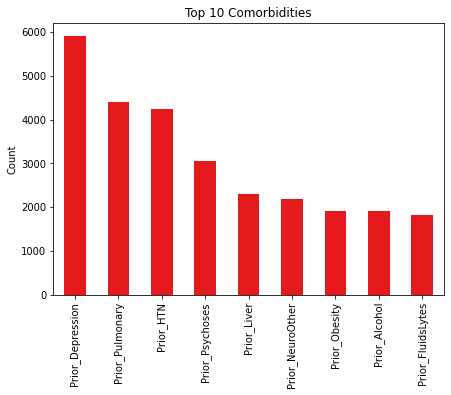

Supplement: S3 Fig — (PNG) [file pone.0278988.s006.png]

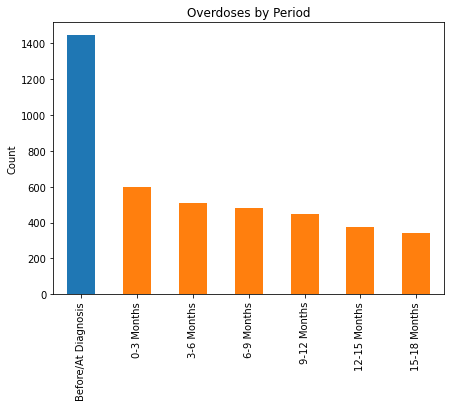

Supplement: S4 Fig — (PNG) [file pone.0278988.s007.png]

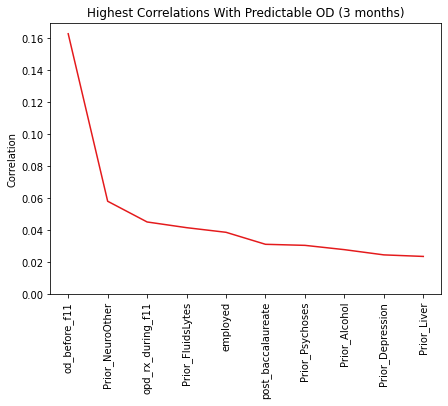

Supplement: S5 Fig — (PNG) [file pone.0278988.s008.png]

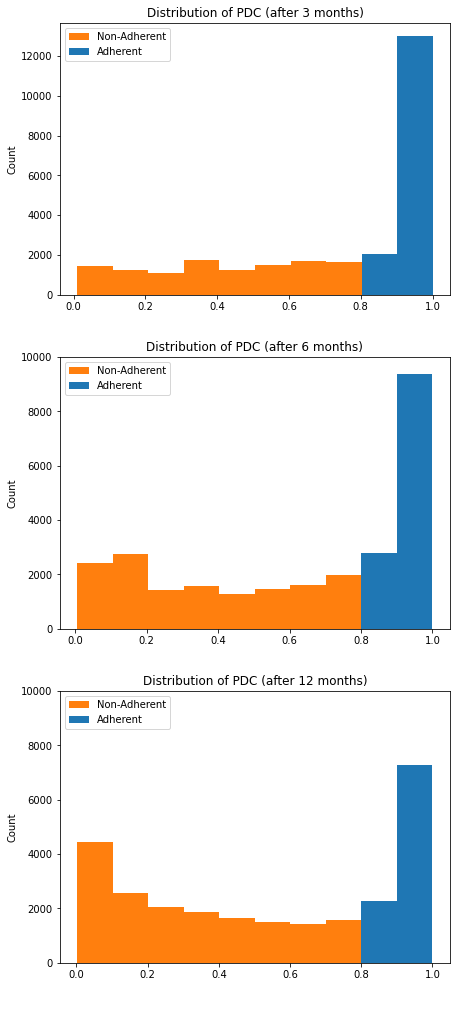

Supplement: S6 Fig — (PNG) [file pone.0278988.s009.png]

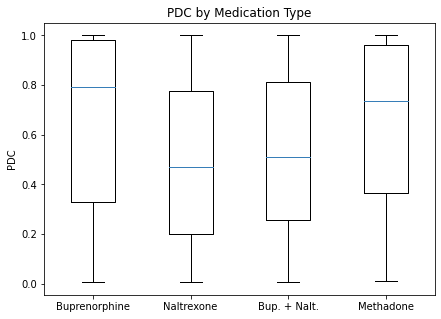

Supplement: S7 Fig — (PNG) [file pone.0278988.s010.png]

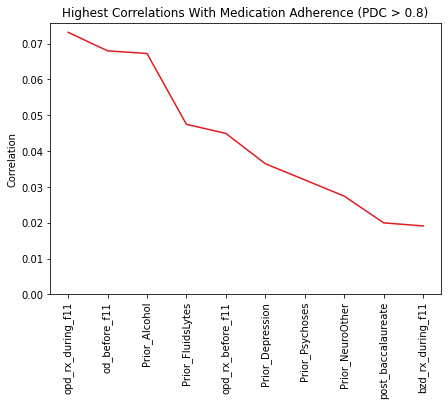

Supplement: S8 Fig — (PNG) [file pone.0278988.s011.png]

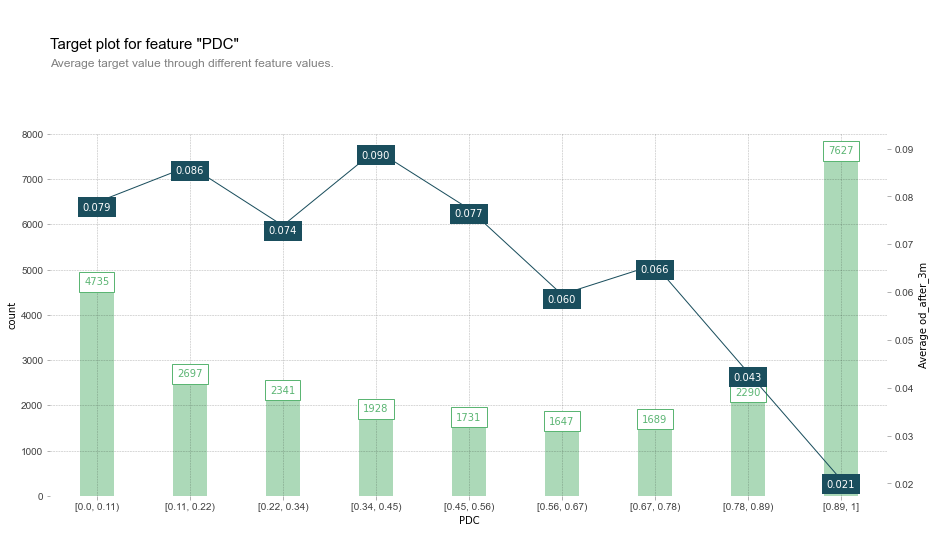

Supplement: S9 Fig — (PNG) [file pone.0278988.s012.png]

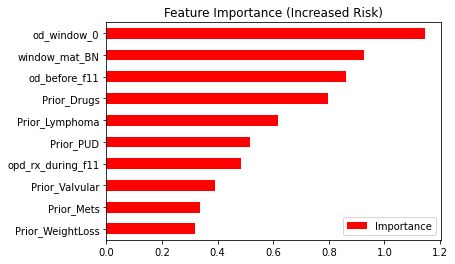

Supplement: S10 Fig — (PNG) [file pone.0278988.s013.png]

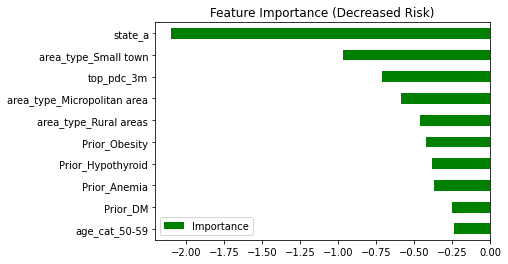

Supplement: S11 Fig — (PNG) [file pone.0278988.s014.png]

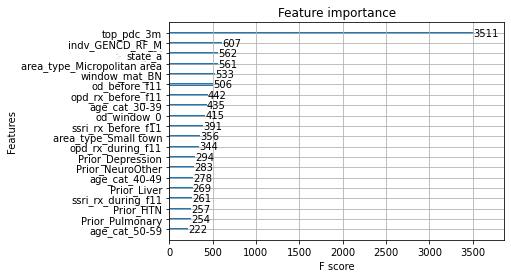

Supplement: S12 Fig — (PNG) [file pone.0278988.s015.png]
